# Expression of Alternative Splice Variants of 6-Phosphofructo-2-kinase/Fructose-2,6-bisphosphatase-4 in Normoxic and Hypoxic Melanoma Cells

**DOI:** 10.3390/ijms22168848

**Published:** 2021-08-17

**Authors:** Sonia E. Trojan, Paulina Dudzik, Justyna Totoń-Żurańska, Piotr Laidler, Kinga A. Kocemba-Pilarczyk

**Affiliations:** 1 Jagiellonian University Medical College, Faculty of Medicine, Chair of Medical Biochemistry, 31-034 Krakow, Poland; sonia.trojan@doctoral.uj.edu.pl (S.E.T.); paulina.dudzik@uj.edu.pl (P.D.); piotr.laidler@uj.edu.pl (P.L.); 2 Jagiellonian University Medical College, Center for Medical Genomics-OMICRON, 31-034 Krakow, Poland; justyna.toton-zuranska@uj.edu.pl

**Keywords:** PFKFB4, isoforms, hypoxia, malignant melanoma, HIF-1

## Abstract

Cancer-specific isoenzyme of phosphofructokinase II (*PFKFB4*), as our previous research has shown, may be one of the most important enzymes contributing to the intensification of glycolysis in hypoxic malignant melanoma cells. Although the *PFKFB4* gene seems to play a crucial role in the progression of melanoma, so far there are no complete data on the expression of *PFKFB4* at the isoform level and the influence of hypoxia on alternative splicing. Using RT-qPCR and semi-quantitative RT-PCR, we presented the *PFKFB4* gene expression profile at the level of six isoforms described in the OMIM NCBI database in normoxic and hypoxic melanoma cells. Additionally, using VMD software, we analyzed the structure of isoforms at the protein level, concluding about the catalytic activity of individual isoforms. Our research has shown that five isoforms of *PFKFB4* are expressed in melanoma cells, of which the D and F isoforms are highly constitutive, while the canonical B isoform seems to be the main isoform induced in hypoxia. Our results also indicate that the expression profile at the level of the *PFKFB4* gene does not reflect the expression at the level of individual isoforms. Our work clearly indicates that the *PFKFB4* gene expression profile should be definitely analyzed at the level of individual isoforms. Moreover, the analysis at the protein level allowed the selection of those isoforms whose functional validation should be performed to fully understand the importance of *PFKFB4* expression in the metabolic adaptation of malignant melanoma cells.

## 1. Introduction

While the incidence of many tumor types is decreasing, the number of melanoma cases has been increasing worldwide over the past decades [1,2], constituting this malignancy the most invasive and aggressive cancer. Although melanoma may be a relatively rare disease, in fact, it ranks at fifth position in men and seventh position in women among the most common malignancies accounting for 5% and 4% of all new cancer cases, respectively [3]. What is more, the prognosis for patients in advanced stages of melanoma and with distant metastases remains poor with a median rate of survival of approximately 9 months and with less than 10% of patients surviving beyond 5 years [4].

Among the main recognized risk factors of melanoma are chronic sun exposure of the skin, personal or family history of melanoma, large numbers of naevi, genetic defects, and immunodeficiency [1,5,6,7,8]. Regardless of the cause, melanoma arises from pigment-producing cells called melanocytes that reside on the basal layer of the epidermis. Although genetic, epigenetic, and functional changes in cancer cells are crucial for the process of carcinogenesis, the increasing number of literature reports highlight the significant role of the skin microenvironment in melanoma initiation and progression.

An important component of the skin microenvironment that can markedly alter the behavior of cells by direct regulation of genes involved in metabolism, survival, angiogenesis, and apoptosis is tissue oxygenation, more precisely, low oxygen partial pressure [9,10,11,12]. Oxygen level in tissues is a result of a balance between oxygen delivery and its consumption [13]. These phenomena are particularly relevant in the skin, where mild hypoxia is a natural state, while in most solid tumors low oxygen (hypoxic) conditions occur only at a certain stage of cancer growth when the tumor mass is insufficiently vascularized. Much of the experimental work on skin has been limited to quantitative evaluation of its oxygenation. Measurements performed either by needle electrodes or hypoxia-specific probes revealed the gradient of oxygen levels from the value of 10% O_2_ for the dermis, to 0.5–1% O_2_ for the superficial region of skin [11,12,13,14]. Notably, since melanocytes reside in the dermal-epidermal junction they are therefore constantly exposed to a hypoxic environment [12]. What is more, literature reports demonstrate that about 50–60% of advanced solid tumors, including melanomas, are characterized by areas of severe hypoxia with the oxygen concentration ranging from 0.5 to 1.5% of O_2_ [9,10,13].

Several pathways have been identified to mediate the adaptive response of cells to hypoxia among which HIF-1, belonging to the hypoxia-inducible factor (HIF) family of transcription factors, is a primary mediator. Importantly, our recent studies [15] have shown that the majority of analyzed HIF-1 targeted genes involved in glucose breakdown show high expression in normoxic (21% O_2_) as well as hypoxic (1% O_2_) conditions indicating that melanoma cells exhibit high basal glycolytic profile regardless of the oxygen content. Interestingly, the only gene, which expression was strongly altered in hypoxic (1% O_2_) conditions was *PFKFB4*, the one coding for the cancer-specific isoenzyme of phosphofructokinase II (PFK-II). This indicates that induction of *PFKFB4* gene expression can be considered a crucial mechanism behind glycolysis enhancement in hypoxic melanoma cells. What is more, our recent analysis revealed that high *PFKFB4* expression contributes to the poor prognosis of malignant melanoma patients and correlates with significantly worse distant metastasis-free survival and shorter overall survival [15]. So far only very limited information related to the expression and the role of *PFKFB4* isoenzyme in melanoma has been collected. Minchenko et al. in 2005 reported the expression of two isoforms of *PFKFB4* isoenzyme in a single human melanoma cell line DB-1 [16]. In addition, the authors also reported that the expression of *PFKFB4* was induced by hypoxia via HIF-1 dependent mechanism. Our current study provides novel information about additional *PFKFB4* isoforms and alternative splicing events that may be crucial for cancer-specific metabolic changes in melanoma cells.

## 2. Results

### 2.1. The Response of Malignant Melanoma Cells to Low Oxygen Concentration

**Figure 1:**
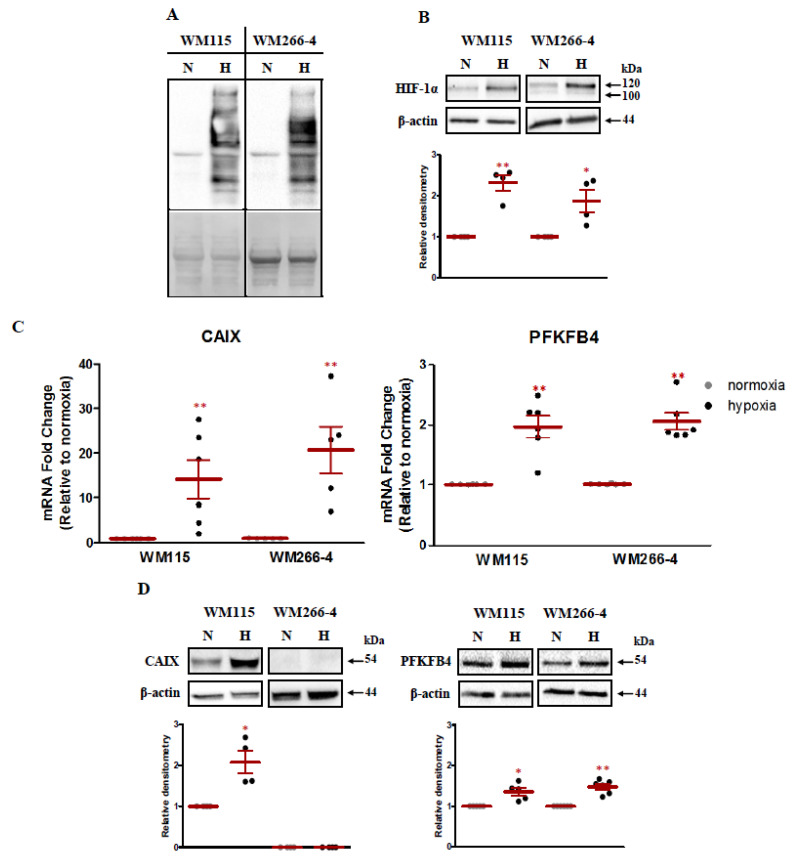
The response of malignant melanoma cells to low oxygen concentration. (**A**) Upper panel: Malignant melanoma lines, WM115 and WM266-4, were cultured with 100 µM pimonidazole (hypoxyprobe) in normoxic (N) and hypoxic conditions (H) for 16 h. The formation of pimonidazole–protein adducts were detected using Western Blot. Lower panel: Ponceau S stained membrane is shown as an internal control for equal protein loading. (**B**) Upper panels: Melanoma cells were cultured for 16 h in normoxia and hypoxia. Then HIF-1 alpha subunit accumulation was verified using the Western Blot. β-actin is shown as an internal control for equal loading. Lower panels: Densitometry analysis of Western Blot bands intensity normalized to β-actin. Relative densitometry value is the average of four independent experiments. The mean ± SEM is shown. Student’s *t*-test was used to evaluate the influence of hypoxia on HIF-1 alpha subunit stabilization. * *p* < 0.05 by Student’s *t*-test, ** *p* < 0.01 by Student’s *t*-test, *p*-value between 0.05 and 0.1 by Student’s *t*-test was given as an indication of the trend. (**C**) *CAIX* and *PFKFB4* expression was analyzed by RT-qPCR in both melanoma cell lines under hypoxic and normoxic conditions. Expression data for each transcript was normalized to that for the reference gene TBP. Means ± SEM of at least five independent experiments are presented relative to expression in normoxic controls. The Student *t*-test was used to evaluate the differences between normoxic and hypoxic expression of *CAIX* and *PFKFB4*. ** *p* < 0.01 by Student’s *t*-test. (**D**) Upper panels: Melanoma cells were cultured for 16 h in normoxia and hypoxia. Then CAIX and PFKFB4 expression was verified using the Western Blot. β-actin is shown as an internal control for equal loading. Lower panels: Densitometry analysis of Western Blot bands intensity normalized to β-actin. Each relative densitometry value is the average of at least four independent experiments. The mean ± SEM is shown. Studen’st *t*-test was used to evaluate the influence of hypoxia on CAIX and PFKFB4 expression. * *p* < 0.05 by Student’s *t*-test, ** *p* < 0.01 by Student’s *t*-test, *p*-value between 0.05 and 0.1 by Student’s *t*-test was given as an indication of the trend.

Two melanoma cell lines have been chosen for our analysis: WM115 from the vertical growth phase and its metastatic derivative WM266-4. The cells were cultured simultaneously under normoxic (21% O_2_) and hypoxic (1% O_2_) conditions for 16 h. To investigate the effect of hypoxia on *PFKFB4* gene expression first we have validated our experimental setup. In the first step, the oxygen tension within the cells was assessed using the pimonidazole compound. In hypoxic conditions, pimonidazole forms adducts with intracellular proteins, which can be furthered visualized by Western Blot. As shown in Figure 1A we have observed the intense staining for protein–pimonidazole adducts in hypoxic cells in comparison to normoxic cells, for which no product visualization was observed.

As HIF-1 can be considered as the primary mediator of the adaptive response of cells to hypoxia, we have examined the impact of low oxygen concentration on the stabilization of HIF-1 alpha subunit protein. As presented in Figure 1B, hypoxia-induced accumulation of HIF-1 alpha. To determine whether HIF-1 accumulation triggers a transcriptional response, using TaqMan RT-qPCR, we have studied the mRNA expression level of Carbonic Anhydrase IX (*CAIX*) in parallel to *PFKFB4*. *CAIX* is a HIF-1 target gene [17,18] and expression of this gene in many types of tumors indicates its relevance as a general marker of tumor hypoxia [19]. Moreover, the induction of the *CAIX* gene was the most apparent among 12 HIF-1 triggered genes out of 24 tested at mRNA level in both melanoma cells lines (data not shown). The number of reports in the literature indicates that *CAIX* plays a role in many processes related to carcinogenesis [19,20,21,22] and seems to be crucial in adaptation to extracellular acidosis of cancer cells, including melanoma [23,24]. Moreover, the expression of *CAIX* significantly contributes to the poor progression of melanoma patients [22]. Furthermore, recent studies by Min-Chien Hsin et al. revealed that *CAIX* overexpression in cervical cancer cells increases *PFKFB4* expression and regulated epithelial–mesenchymal transition, promoting cancer cell migration [25]. What is more, cervical cancer patients, that exhibit high both *CAIX* and *PFKFB4* expression demonstrate shorter overall survival [22]. Our analysis revealed that under hypoxic conditions the induction of *CAIX* was much stronger than induction of *PFKFB4*. We have observed a 14.15 and 20.72 fold change of *CAIX* expression under hypoxic conditions in WM155 and WM266-4 cell lines, respectively, at mRNA level (Figure 1C). The obtained data were in accordance with our previous research [15]. The response to low oxygen concentration was much weaker for the *PFKFB4* gene. An approximately two-fold increase in *PFKFB4* expression in hypoxia in both melanoma cell lines was observed (Figure 1C).

The obtained results were furthered confirmed by the examination of the influence of low oxygen concentration on CAIX and PFKFB4 protein expression (Figure 1D). For CAIX in WM115 melanoma cell line, we have observed the 2.09 fold induction of expression under hypoxic conditions. In WM266-4 cell the analysis showed no CAIX protein expression both under normoxic and hypoxic conditions. Since 16-hour incubation of cells in the hypoxic condition could not have been sufficient for the observation of an increase in the level of CAIX protein expression, we tested whether the longer time of hypoxia stimulation (48 h) will increase the expression of CAIX protein. Our additional analysis revealed that after 48 h of hypoxia, the induction of CAIX protein expression, in comparison to 16 h, has not changed (data not shown). The same trend was observed for PFKFB4, as the expression of the protein was comparable regardless of the duration of hypoxia stimulation of melanoma cells (data not shown). The induction of PFKFB4 expression after 16 h was on average 1.36 (WM115) and 1.48 (WM2664) higher under hypoxic conditions as compared to normoxia. Our analysis shows that hypoxia does not significantly regulate PFKFB4 expression at the protein level, while at the mRNA level the response is much weaker than for CAIX. Taking into account the above data, we assume that the induction may be isoform-specific and perhaps seen in the expression of other transcripts.

### 2.2. The Differences in Structure and Expression of Six Isoforms of PFKFB4 Isoenzyme

**Figure 2:**
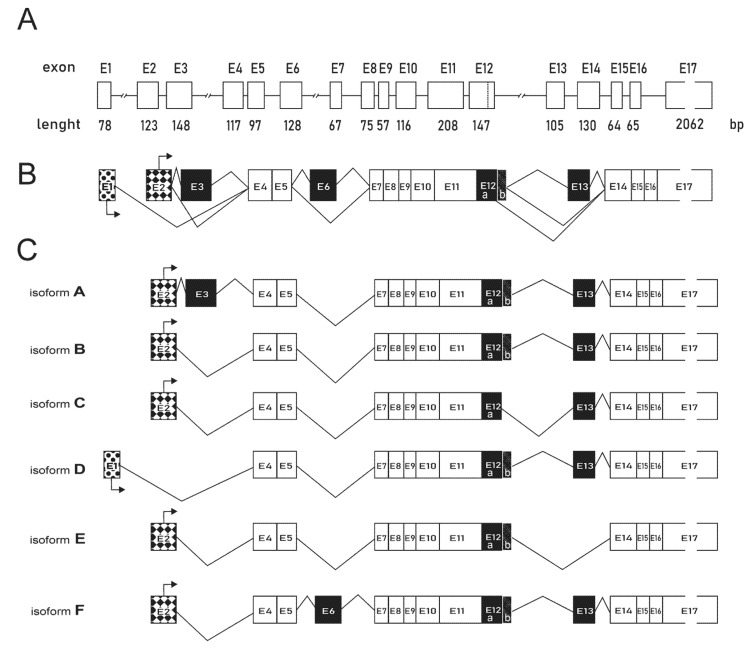
*PFKFB4* gene structure and alternative splicing isoforms. (**A**) Schematic representation of the exon-intron organization of the *PFKFB4* gene. (**B**) Alternative splicing of *PFKFB4* pre-mRNA. Constitutive exons, present in all final mRNAs are shown in white. Alternative cassette exons that may be included or excluded are shown in black. Alternative promoters are patterned. (**C**) Comparison of six *PFKFB4* isoforms resulting from alternative splicing. The mRNA sequences were obtained from the NCBI Gene database with the following codes: isoform A (NM_001317134.2), isoform B (NM_004567.4), isoform C (NM_001317135.2), isoform D (NM_001317136.2), isoform E (NM_001317137.2), and isoform F (NM_001317138.2).

According to the OMIM NCBI Gene database, six isoforms of *PFKFB4* can be formed, which were all captured by the primers used in our TaqMan analysis for the *PFKFB4* expression. Therefore, in the next step, we have examined the contribution of individual isoforms to the final *PFKFB4* gene expression, First, using the OMIM NCBI Gene database we have compared the sequences of six (A–F) *PFKFB4* isoforms. In our analysis, we compared all isoforms with respect to the canonical isoform B, which was first discovered in 1999 by Manzano and coworkers who have provided the evidence for a new PFK-2/FBPase-2 gene coding for a human testis isozyme [26]. The differences in the structure of individual isoforms, resulting from the process of alternative splicing, are shown in Figure 2. All the exons were numbered according to the order of their occurrence in the gene sequence. According to the analysis, isoform D contains an alternative promoter. For isoforms A and F, an additional cassette exon is included exon 3 and exon 6, respectively, while in isoforms C and E, the exclusion of cassette exon 12b and 13, respectively, were observed.

**Figure 3:**
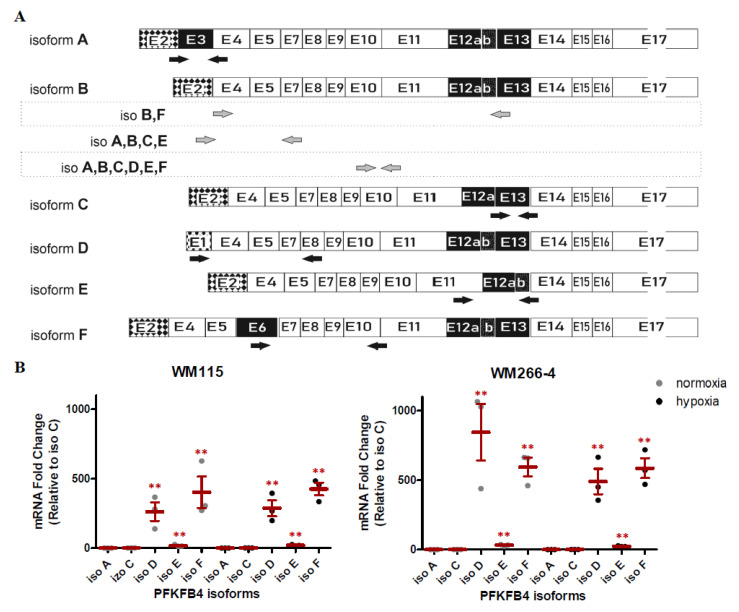

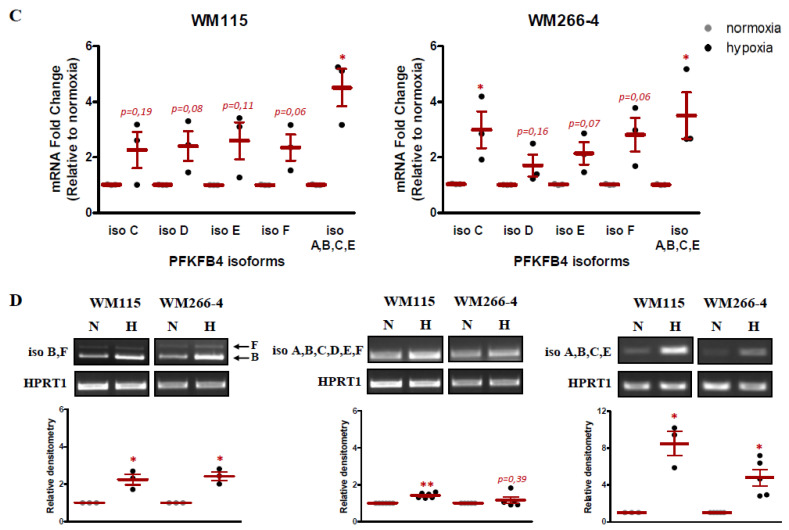
Expression of PFKFB4 isoforms in normoxic and hypoxic conditions. (**A**) The binding position of primers specific to PFKFB4 isoforms. (**B**) Expression of PFKFB4 isoforms: A, C, D, E, and F under normoxic and hypoxic conditions. The expression levels of all isoforms were normalized with respect to isoform C and the fold change values were calculated. The mean ± SEM is shown of three independent experiments. Student’s *t*-test was used to evaluate the statistical significance. * *p* < 0.05 by Student’s *t*-test, ** *p* < 0.01 by Student’s *t*-test. (**C**) The influence of 16 h hypoxia on indicated isoforms expression were assessed using RT-qPCR. Expression data for each transcript was normalized to that for the reference gene β-actin, then the expression level of each isoform under hypoxia was normalized with respect to normoxia, and the fold change values were calculated. The mean ± SEM is shown of three independent experiments. Student’s *t*-test was used to evaluate the statistical significance. * *p* < 0.05 by Student’s *t*-test, ** *p* < 0.01 by Student’s *t*-test, *p*-value between 0.05 and 0.1 by Student’s *t*-test was given as an indication of the trend. (**D**) The influence of hypoxia on the expression of PFKFB4 isoforms including isoform B. The expression levels of indicated PFKFB4 isoforms were assessed using semi-quantitative RT-PCR. HPRT1 was used as an internal control for equal loading. Lower panel: Densitometry analysis of semi-quantitative RT-PCR bands intensity normalized to HPRT1. Each relative densitometry value is the average of at least three independent experiments. The mean ± SEM is shown. Student’s *t*-test was used to evaluate the influence statistical significance. * *p* < 0.05 by Student’s *t*-test, ** *p* < 0.01 by Student’s *t*-test, *p*-value between 0.05 and 0.1 by Student’s *t*-test was given as an indication of the trend.

To analyze the relationship between individual isoforms in melanoma cells, we have compared their expression levels under normoxic conditions, using SYBR™ Green Master Mix (ThermoFisher, Waltham, MA, USA). For each isoform (except B) we have designed the individual pair of primers (Figure 3A). As isoform B, the canonical sequence does not represent any unique parts, there was no possibility to design specific primers capturing this isoform only. In each examined melanoma cell line, we have observed the expression of four out of five tested *PFKFB4* isoforms (C,D,E,F), whereas the expression of isoform A was not detected. As the isoform C represented the lowest level of expression, we compared the expression levels of other expressed isoforms with respect to this one. The expression of isoform C was normalized to 1, and the expression of the remaining isoforms was presented in Figure 3B as the fold change. The analysis shows that the isoforms with the highest expression are D and F, both in WM115 as in the WM266-4 cell line. Under hypoxic conditions, the overall expression profile of the individual isoforms remained unchanged.

### 2.3. The Effect of Hypoxia on PFKFB4 Isoforms Expression in Melanoma Cell Lines

Next, using Real-time PCR based on SYBR^TM^ Green, we have examined the influence of low oxygen concentration on expression levels of *PFKFB4* isoforms (Figure 3C). For the WM115 cell line, we have observed an approximately two-fold increase in the expression level of each studied isoform. For the WM266-4 cell line, the results were similar to those obtained for WM115, with the exception of isoforms C and F, for which a greater induction in hypoxia was observed, amounting to 2.99 and 2.82 fold-change, respectively. Although most of the observed changes were not statistically significant, the obtained trend was clear. Perhaps the lack of stability in the level of induction requires a greater amount of analysis to achieve statistical significance.

To fully visualize the influence of low oxygen concentration on the expression of *PFKFB4* isoforms, we have also investigated the influence of hypoxia on the level of isoform B. As mentioned earlier, since isoform B (a constitutive one) cannot be analyzed individually, we decided to design an additional pair of primers, allowing for indirect analysis. Given the significant level of expression of the D and F isoforms, which could mask the influence of hypoxia on the expression of the B isoform, we excluded these two isoforms from further analysis designing primers specific for isoforms ABCE (Figure 3A). The obtained results showed that for the WM266-4 melanoma cell line the increase in expression of isoforms mix (ABCE) in hypoxia was significant and greater than for the individual isoforms, and was equal to 3.51 fold increase (Figure 3C). However, for the WM115 cells, the increase in induction in hypoxia was even higher, with a 4.51 fold-change (Figure 3C). The significant increase in expression, under hypoxic conditions, for the isoforms mix compared to the single isoforms, may suggest that isoform B is the one with the strongest induction of expression under hypoxia. To analyze the hypoxic regulation of isoform B, we designed primers specific for two isoforms, B and F (Figure 3A), and conducted a semi-quantitative RT-PCR reaction, allowing for visualization of isoform B on the agarose gel electrophoresis (Figure 3D, left panel). The designed primers allowed for the observation of changes in expression derived from isoform F and B as two separate products of 1039 and 911 bp length respectively (Figure 3D, left panel). Our studies confirmed that isoform B shows significant expression induction under hypoxic conditions, in comparison with unexposed cells. The observed effect was similar in both melanoma and lines, with a 2.25 increase for WM115 and 2.43 for the WM266-4 cell line. For comparative purposes, using the same technique we have examined the expression level of the *PFKFB4* gene using primers capturing all six isoforms as well as the isoforms mix after subtraction of the D and F isoforms (Figure 3A). As shown in Figure 3D (middle panel), the densitometry analysis showed the 1.42 and 1.17 fold increase of all *PFKFB4* isoforms under hypoxic conditions in WM115 and WM266-4 cell lines, respectively. The obtained results represent the same trend as observed for TaqMan analysis (Figure 1C). As for the mix of A,B,C,E isoforms (Figure 3D, right panel), in both melanoma cell lines, we have observed the induction of expression under hypoxia, with 8.51 and 4.80 increase for WM115 and WM266-4 respectively and, these results also confirmed the data from SYBR Green analysis (Figure 3C). The obtained results revealed that although isoform B exhibits significant expression induction under hypoxic conditions, the changes in its expression do not reflect the expression level of the entire *PFKFB4* gene, because it may not be the isoform with the highest abundance.

### 2.4. The Effect of Alternative Splicing on PFKFB4 Protein Sequence

**Figure 4:**
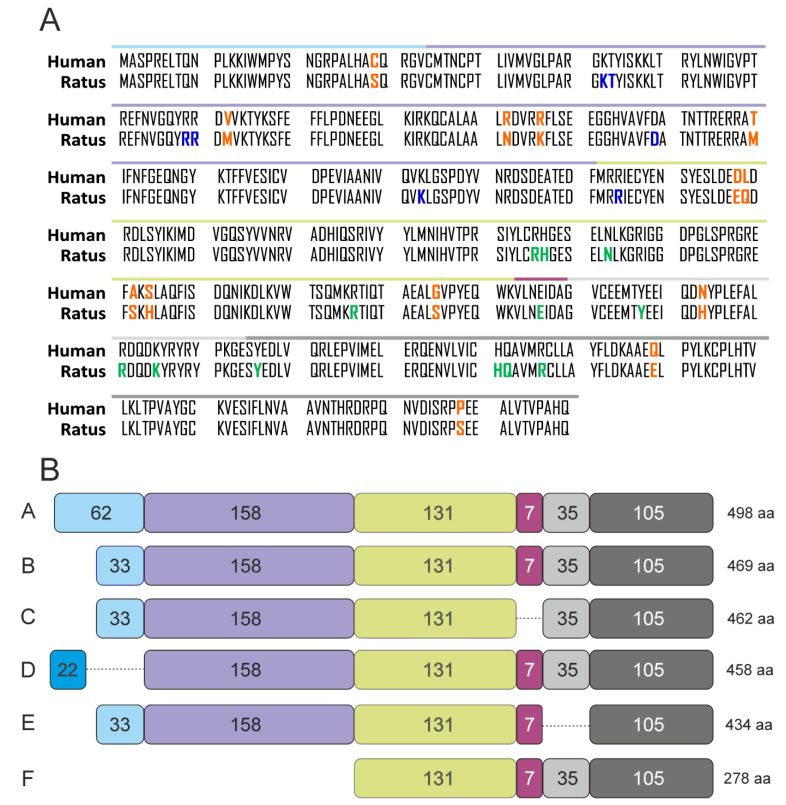
Isoforms of PFKFB4 protein. (**A**) Comparison of the PFKFB4 protein sequence of humans (NCBI Reference Sequence: NP_004558.1) and *Rattus Norvegicus* (NCBI Reference Sequence: NP_062206.1). Differences in amino acid sequence are marked in orange. Amino acids of the 6-PF-2-K active site were colored in blue and amino acids of the Fru-2,6-P2ase active site were colored in green. Based on the Hasemann et al. Kinase domain from 30th to 249th amino acid, phosphatase domain from 252nd to 438th amino acid. Colored lines above the one-letter amino acid abbreviations reflect corresponding parts of protein structure presented in panel B (in relation to isoform B). (**B**) Schematic representation of PFKFB4 protein isoforms. The amino acid sequences were obtained from the NCBI Protein database with the following codes: isoform A (NP_001304063.1), isoform B (NP_004558.1), isoform C (NP_001304064.1), isoform D (NP_001304065.1), isoform E (NP_001304066.1), isoform F (NP_001304067.1).

**Figure 5:**
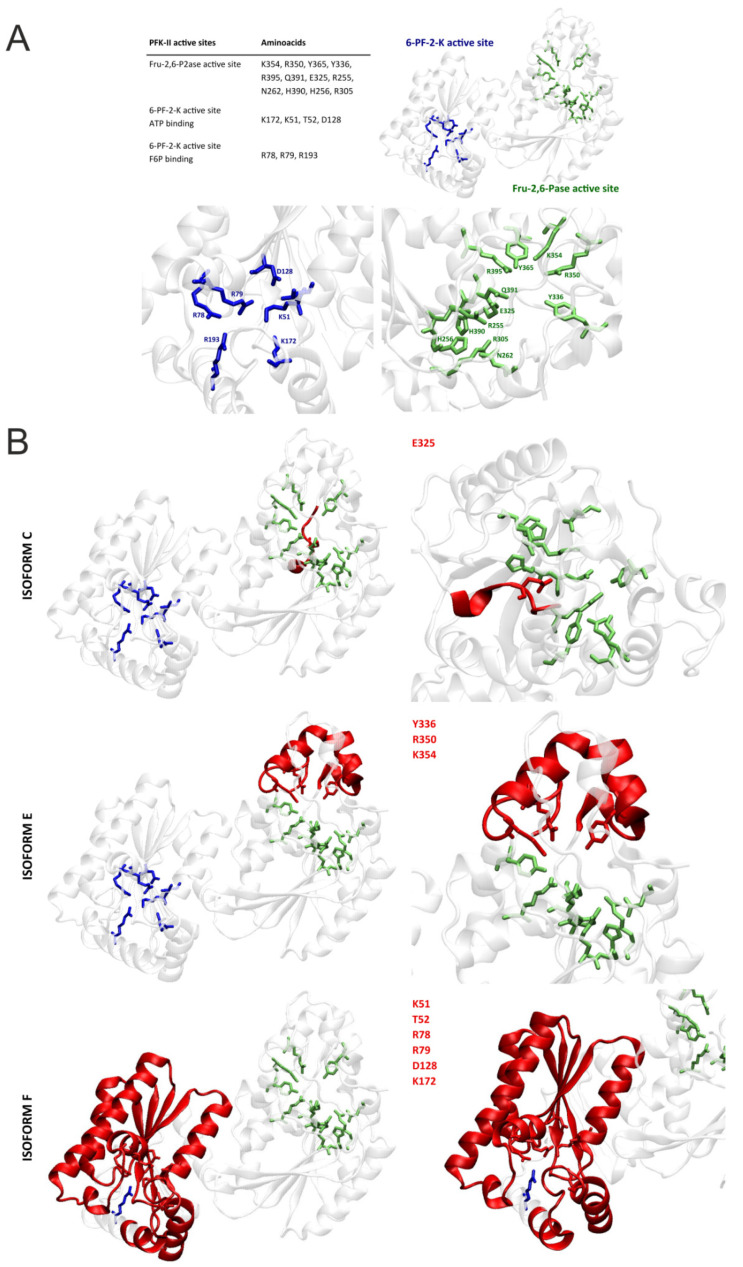
Visualization of PFKFB4 protein and its catalytic domains. (**A**) Left upper panel: Key amino acids of 6-PF-2-K and Fru-2,6-P2ase active sites. Right upper panel: Structure of *Rattus Norvegicus* PFKFB4 protein. The structure was obtained from the Protein Data Bank database with the following code: 1BIF. The protein was visualized using VMD software. The visualized sequence starts from the 38th amino acid (cysteine). The alternative start of the isoforms A and D are not considered in the structure. Lower panel: Binding pockets of 6-phosphofructo-2-kinase/fructose-2,6-bisphosphatase. Amino acids of the 6-PF-2-K active site were colored in blue and amino acids of the Fru-2,6-P2ase active site were colored in green. Based on the Hasemann et al. (**B**) Graphical representation of PFKFB4 protein isoforms. Amino acids of the 6-PF-2-K active site were colored in blue and amino acids of the Fru-2,6-P2ase active site were colored in green. Amino acids not present in the structure were colored in red. Left panel: view of the whole protein structure. Right panel: Focus on the catalytic centers.

Lastly, we studied the effect of alternative splicing on the PFKFB4 protein isoforms and their potential catalytic activity. So far no crystallographic structure for human PFKFB4 is available. For our analysis, we utilized the X-ray structure of the Rattus norvegicus testes PFKFB4 (PDB ID: 1BIF), as it shows more than 97% of similarity in amino acid sequence (Figure 4A) and was successfully used in the literature for comparative purposes [27,28]. As can be seen in Figure 5A, based on the work of Hasemann and others [29] we have in particular, taking into account the amino acids constituting the catalytic centers of the kinase and phosphatase domains. Next, using the NCBI Protein Database, we have compared the structures of six PFKFB4 protein isoforms. The graphical representation of the similarities and differences for the structures has been shown in Figure 4B. As can be seen, isoforms A, B, and D show high sequence similarity, and slight differences at the beginning of the sequence do not alter the structure or functionality of the protein. While isoforms C and E exhibit some deficiencies of 7 and 35 amino acids respectively, in comparison to the canonical structure of isoform B. The lack of this part of the structure is associated with the omission of fragments containing amino acids essential for the activity of the catalytic center of the phosphatase domain (iso C: E325; iso E: Y336, R350, and K354) (Figure 5B). The omission of relevant exons will probably be associated with an alternative folding or unfolding of the phosphatase domain most likely associated with impairment of catalytic ability of this function of the bifunctional enzyme. In the case of the F isoform, due to the delayed start of translation, this isoform does not contain/comprise a kinase domain in its structure, so, if only properly folded, it most likely exhibits only phosphatase activity. Based on the presented data we presume that because the isoforms with the highest expression in melanoma cells are the D and F isoforms, the cells may demonstrate not only a functional PFKFB4 protein but also increased phosphatase activity due to the presence of the F isoform.

## 3. Discussion

The crucial function of 6-phosphofructo-2-kinase/fructose-2,6-bisphosphatase (PFKFB) is to modulate the cellular levels of fructose-2,6-bisphosphate, the key molecule responsible for the regulation of glucose flux through two alternative pathways, the pentose phosphate pathway (PPP) and glycolysis. The PFK-II enzyme contains both kinase and phosphatase domains in its structure. The activity of the kinase domain leads to an increase in the level of fructose 2,6-bisphosphate, directing glucose flow into the glycolysis pathway. The activity of the phosphatase domain, through dephosphorylation of the key activator of glycolysis, will stimulate the pentose phosphate pathway [30]. Both of these pathways are important for cancer cells as PPP generates the majority of NADPH, providing reducing power for the reductive biosynthesis of macromolecules, including nucleic acid synthesis, and preventing oxidative stress by scavenging ROS [31]. On the other hand, enhanced glycolytic rate provides cellular energy as well as metabolic intermediates for macromolecular synthesis [30]. Thus, for neoplastic cells, both activities of cancer-specific PFK-II isoenzymes (*PFKFB3, PFKFB4*), although opposing, can significantly stimulate tumor progression [28]. This is especially important in melanoma cells, as this neoplasm develops in an environment of reduced partial oxygen pressure [12]. Hence, the only possibility of ATP synthesis is via glycolysis, in which high activity determines the survival and proliferation of cancer cells by providing intermediates necessary for biosynthesis. On the other hand, PPP activity allows for cell protection against ROS, which is generated in large amounts during the cyclic hypoxia/re-oxygenation process, which is a hallmark of tumors developing in a hypoxic environment, such as melanomas. In addition, the oxidative arm of PPP constitutes the cell’s key source of NADPH, which is crucial for reductive biosynthesis, while the glycolysis intermediates allow for ribose-5-phosphate synthesis in the non-oxidative PPP arm [28,30,31,32]. Therefore, not only the regulation of expression of cancer-specific *PFKFB3* and *PFKFB4* isoforms but also the regulation/modulation of the activity of two opposing domains will be crucial for the tumor progression [32,33]. So far, the study performed by Chesney et al. has shown that PFKFB3 and PFKFB4 exhibit the dominant activity of the kinase domain. When recombinant PFKFB4 and PFKFB3 were tested, the kinase/phosphatase activity ratios were estimated at 4.3:1 and 81.7:1, respectively [34]. Studies by Ros et al. indicate that the phosphatase domain of PFKFB4 may also be important for prostate cancer cells, as PFKFB4 silencing increased oxidative stress and lowered the NADPH level resulting from the lower activity of the PPP pathway [35]. Nevertheless, since the posttranslational modification is crucial for PFKFB3/PFKFB4 enzyme activity the final kinase/phosphatase ratio may be dependent on the cellular context [36]. Another important aspect in addition to the regulation of domain activity at the level of a single isoenzyme is the regulation of expression at the level of isoforms. Especially that the activity can be easily controlled at the level of isoforms that possess kinase or phosphatase domain only. The following work focuses on the analysis of the expression of isoforms produced from the *PFKFB4* gene under both hypoxic and normoxic conditions. The PFKFB4 isoenzyme was first described by Mazano et al., who isolated cDNA from the human testis cDNA library [26]. Subsequently, sequencing revealed that the ORF is 1407 nucleotides, which encodes a protein of 469 amino acids. The isoform described by Mazano is one of the six isoforms currently available in the NCBI database—so-called isoform B (NM_004567.4), which is considered to be the canonical isoform of *PFKFB4*. When it comes to the expression of the remaining *PFKFB4* isoforms in melanoma, Michenko et al. only demonstrated that an additional *PFKFB4* isoform is expressed in the DB-1 melanoma line with 148-bases insert in the 5’-region [16]. The isoform described in the DB-1 line corresponds to the A isoform in the NCBI database and, as Michenko showed, its expression, similar to the canonical B isoform, is controlled by the HIF-1 factor. Our research has shown that five isoforms of *PFKFB4* (B, C, D, E, F) are expressed in the tested melanoma cell lines (WM266-4 and WM115). Our studies revealed that out of the four expressed isoforms that could be analyzed with specific primers (C, D, E, F), isoforms D and F represented the highest expression level. It is also worth noting that the induction of gene expression in hypoxia, at the level of all isoforms, was rather insignificant compared to the induction observed for the group of B, C, E isoforms. As isoforms C and E did not show a significant induction in hypoxia, isoform B was most likely responsible for the observed effect, however, this isoform could not be analyzed as an independent isoform in the RT-qPCR analysis. Our hypothesis seems to be confirmed by the result of semi-quantitative PCR, indicating that isoform B is clearly induced under hypoxic conditions. As for the highest expressed isoforms D and F, it can be assumed that they cover changes in expression levels of isoform B, and thus the influence of hypoxia is much more clear/marked when the group of B, C, E isoforms is analyzed compared to the change in gene expression at the level of all expressed isoforms (B, C, D, E, F). It is also worth noting that isoform D, like the B isoform, contains both kinase and phosphatase domains, in contrast to isoform F, which comprise only a phosphatase domain. Presumably, isoform D may be considered as a constitutive isoform, in which basal expression in normoxia is high, while isoform B may constitute the major hypoxia-inducible isoform. As for the F isoform, it would be crucial to verify its expression at the protein level, as it only contains a phosphatase domain, so its enzymatic activity would shift glucose consumption towards the PPP pathway. Our work has shown that the analysis of the influence of hypoxia on the expression of the *PFKFB4* gene depends on the selection of primers and isoforms that are subject to analysis. In most of the studies where *PFKFB4* expression has been analyzed, primers including all the described isoforms have been used [34,37,38,39,40,41]. Such an approach does not allow for the analysis of the expression of particular isoforms, among others, the isoform considered as the constitutional isoform of *PFKFB4*. Moreover, there is no possibility of examining the constitutive isoform by the RT-qPCR method, and when it comes to melanoma cells, using primers covering all six isoforms, most probably isoforms with the highest expression (D and F) significantly influence the final picture of *PFKFB4* expression pattern. As for the analysis at the protein level, it is also impossible to determine whether the apparently visible expression of *PFKFB4* is the expression of the isoform B, so far considered constitutive, or isoform D, as the difference in the molecular weight does not allow for the separation of the two isoforms in classical Western Blot analysis. With regard to the remaining isoforms expressed in the studied melanoma cells, (C, E, and F), it should be emphasized that isoforms E most likely is phosphatase dead, due to the lack of amino acids essential for the proper activity of the phosphatase domain. The conclusion isn’t so clear for isoform C. A loop proceeds a short deleted region. This loop could in principle substitute the deleted region in the protein fold. Especially that it contains another glutamic acid. This is speculative though. When it comes to isoform F, it only possesses the phosphatase domain in its structure. In addition, the C and E isoforms show low expression compared to the D and F isoforms. Therefore, it seems that for melanoma cells, constitutive, high expression of the D and F isoforms as well as the expression of the canonical B isoform may be of key importance. It would be crucial to determine whether isoform B or D constitute the canonical isoform of *PFKFB4* in melanoma cells. This could theoretically be determined by specific silencing of the D isoform and subsequent protein analysis of PFKFB4 expression using the Western-blot method. As for the isoform F, it would be essential to resolve whether this isoform is expressed at the protein level. In general, our results open a new area for more functional studies of which the most interesting would be the analysis of the effect of particular isoforms overexpression on melanoma cell phenotype using for example expression plasmids. Our work has shown that only isoform-specific expression analysis provides a complete picture of changes in *PFKFB4* levels in melanoma cells. This is especially important if the final phosphatase or kinase activity is regulated at the level of isoforms expressed in a specific tumor. In conclusion, our study pointed a need for proper analysis of *PFKFB4* isoforms to understand the complex process of metabolic changes in cancer. On the other side, recent studies have shown that PFKFB4 is not only involved in glucose metabolism but may also regulate other processes crucial for carcinogenesis. For instance, studies by Dasgupta et al. [42] have shown that PFKFB4 can phosphorylate transcriptional coactivator SRC-3, which results in promoting glucose flux, the same contributing to enhanced proliferation of breast cancer cells. PFKFB4, by interacting with Etk, promotes small lung-cancer chemoresistance through the regulation of autophagy [43,44]. What is more, recent studies by Sittewelle et al. have identified a novel, glycolysis-independent function of PFKFB4, which promotes ICMT-RAS interactions, which results in activation of AKT signaling and enhancing melanoma cell migration [45]. To sum up, our study highlights important differences in mRNA splicing between *PFKFB4* isoforms expressed in melanoma cells and provides information about isoforms that should be functionally validated to confirm their role in metabolic adaptation and cellular signaling of malignant melanoma cells, which maybe be crucial for carcinogenesis and response to anti-melanoma therapies.

## 4. Materials and Methods

### 4.1. Cell Lines and Cell Cultures

Two human melanoma cell lines WM115 and WM266-4 were obtained from Rockland Immunochemicals (Limerick, PA, USA). Cells were cultured in RPMI 1640 Media (ThermoFisher Scientific, Waltham, MA, USA) supplemented with 10% fetal bovine serum (EURx, Gdansk, Poland) and 100 units/mL of Penicillin-Streptomycin mix (ThermoFisher Scientific, Waltham, MA, USA). Cells were cultured in normoxic (21% O_2_) and hypoxic (1% O_2_) conditions. Reduced oxygen culture conditions were obtained, as described previously [46]. All the cells were tested negative for mycoplasma using MycoCheckTM Mycoplasma PCR Detection Kit (MoBiTec Molecular Biotechnology, Goettingen, Germany).

### 4.2. RNA Isolation and cDNA Synthesis

The total amount of RNA was extracted from melanoma cells with the use of the Universal RNA Purification Kit (EURx, Poland). The purity and concentration of all isolated RNA samples were determined using NanoDrop ND-1000 Spectrophotometer (NanoDrop Technologies, Wilmington, DE, USA). The synthesis of cDNA from 200 ng of isolated RNA was performed using an NG dART RT kit (EURx, Poland). The obtained cDNA was then diluted 5 times.

### 4.3. Reverse Transcription Polymerase Chain Reaction (RT-PCR)

PCR reaction was carried out using Color OptiTaq PCR Master Mix (2×) (EURx, Poland). The PCR mixture contained 0.6 μL of each 10 μM primers (forward and reverse), 5 μL of Color OptiTaq PCR Master Mix (2×), and 0.8 μL of nuclease-free deionized water. 3 μL of cDNA was added to each PCR mixture. The PCR was performed in Bio-Rad T100 Thermal Cycler (Bio-Rad, Hercules, CA, USA). PCR conditions were as follows: initial denaturation for 5 min at 95 °C. Then 95 °C, 56.7 °C, 72 °C, each for 30 s for 27 cycles. To complete the PCR reaction, 10 min incubation at 72 °C was applied. The PCR products were analyzed on 2% *w*/*v* agarose gels containing ethidium bromide. Housekeeping gene HPRT1 was used for normalization to correct for differences in loading of the cDNAs samples.

### 4.4. Real-Time qPCR

Real-time PCR was performed using TaqMan Fast Advanced Master Mix (ThermoFisher, USA) and validated gene expression assays for PFKFB4 (Hs00894603_m1) and CA9 (Hs00154208_m1). The available PFKFB4 primers covered all six PFKFB4 isoforms, described in the OMIM NCBI Gene database. Expression data for each transcript was normalized to that for the reference gene TBP (Hs00427620_m1), which did not change expression upon exposure to hypoxic conditions. TaqMan cycling conditions were according to the manufacturer’s protocol. The real-time PCR was performed in Bio-Rad CFX96 Touch Real-Time PRC (Bio-Rad, USA). Fold changes compared with controls were determined using the comparative Ct method.

To examine the expression of individual isoforms of the PFKFB4 gene, real-time PCR using PowerUp™ SYBR™ Green Master Mix (ThermoFisher, USA) was performed. All the primers were manually designed using the OMIM NCBI Gene database and Primer-BLAST NCBI Tool [47]. The primer sequences are listed in Table 1.

All the primers were synthesized by Sigma-Aldrich (Saint Louis, MO, USA). For each pair of primers, the primer efficiency was tested through the use of serial dilutions. Verification that the melting curve had a single peak with an observed Tm consistent with the amplicon length was performed for every PCR reaction. Expression data for each transcript was normalized to that for the reference gene β-actin (Qiagen, Hilden, Germany), which also did not change the expression upon exposure to hypoxic conditions. The real-time PCR was performed in Bio-Rad CFX96 Touch Real-Time PRC (Bio-Rad, USA). Ct values from triplicate measurements were averaged, and relative expression levels were determined by the ΔΔCt method, then the fold changes compared with controls were determined.

### 4.5. Western Blot

Western Blot analysis was performed as described previously [48]. Primary antibodies used were as follows: anti-HIF-1 alpha antibody (Cell Signaling Technology, Danvers, MA, USA, cat. no. 3716S, dilution factor 1:1000), anti-CAIX antibody (Bioassay Technology Laboratory, Shanghai, China cat. no. 12F10, dilution factor 1:1000), anti-PFKFB4 (Abcam, Cambridge, UK, cat. no. ab137785, dilution factor 1:2000), anti-B-actin (Sigma-Aldrich, Saint Louis, MO, USA, cat. no. A5441, dilution factor 1:10,000). The polyclonal PFKFB4 antibody was directed against amino acids between 200–469 and has potentially covered all six PFKFB4 isoforms. Secondary antibodies used were as follows: anti-rabbit IgG HRP-linked antibody (Cell Signaling Technology, USA), anti-mouse IgG HRP-linked antibody (Cell Signaling Technology, USA). For visualization of protein expression, the SignalFireTM Elite ECL Reagent (Cell Signaling Technology, USA) was used.

### 4.6. VMD: Visual Molecular Dynamics

The crystallographic structure of Rattus norvegicus testes PFKFB4 was obtained from Protein Data Bank (PDB ID: 1BIF). The protein structure was visualized, and all the pictures of the structure were generated using VMD software [49].

### 4.7. Statistical Analysis

For each experiment performed in reduced oxygen conditions, the control of cells cultured in parallel in normoxia was provided, as a confirmation of expected HIF-1 signaling pathway induction. Densitometry values of Western blot and RT-PCR bands intensity were normalized to β-actin and HPRT1, respectively. Each relative densitometry value was presented as the mean of at least three independent experiments. Student *t*-test was performed to evaluate the significance of differences between the level of expression of PFKFB4 isoforms. For the purpose of analysis the *p* values  <  0.05 were considered statistically significant, whereas *p* values between 0.05 and 0.1 were considered as an indication of the trend. Statistical analysis was performed by GraphPad Prism v5.0 software (GraphPad Software Inc., San Diego, CA, USA).

## Figures and Tables

**Table 1:** PCR primer sequences.

PFKFB4 Isoform	NCBI Gene ID	Forward and Reverse Primers	Product Length
Isoform A	NM_001317134.2	F: CAGCGCGGTGAACTTTCAAA	168 bp
		R: GTCATGCACAATGTCCCGGG	
Isoform C	NM_001317135.2	F: CAGTGGAAGGGCGTCTGTG	126 bp
		R: GTCCTCGTAGGACTCCCCTT	
Isoform D	NM_001317136.2	F: TGGACAGAGGCTCGTTAGGA	383 bp
		R: CGCTCTCCGTTCTCGGGTG	
Isoform E	NM_001317137.2	F: TCAAGATCATGGATGTGGGC	351 bp
		R: CCTCGTAGGACGCATCGATC	
Isoform F	NM_001317138.2	F: AATCTAAGCCCATCCACCGC	386 bp
		R: GGACAGGTCCCTATCCAGGT	
Isoforms A, B, C, D, E, F		F: TCAAGATCATGGATGTGGGC	214 bp
		R: TCTTGGCAAACTCCCTGC	
Isoforms A, B, C, E		F: CAGCGCGGTGTGTGCATGAC	233 bp
		R: GCACACTGCTTCCTGATTTT	
Isoforms B and F	NM_004567.4	F: CAGCGCGGTGTGTGCATGAC	Iso B 911 bp
	NM_001317138.2	R: TCACAGACGCCCGCATCGATC	Iso F 1039 bp

## Data Availability

Not applicable.

## References

[1] Rastrelli M., Tropea S., Rossi C.R., Alaibac M. (2014). Melanoma: Epidemiology, risk factors, pathogenesis, diagnosis and classification. In Vivo.

[2] MacKie R.M., Hauschild A., Eggermont A.M.M. (2009). Epidemiology of invasive cutaneous melanoma. Ann. Oncol..

[3] Heistein J.B., Acharya U. Malignant Melanoma. StatPearls.

[4] Lenggenhager D., Curioni-Fontecedro A., Storz M., Shakhova O., Sommer L., Widmer D.S., Seifert B., Moch H., Dummer R., Mihic-Probst D. (2014). An Aggressive Hypoxia Related Subpopulation of Melanoma Cells is TRP-2 Negative. Transl. Oncol..

[5] Ward W.H., Lambreton F., Goel N., Yu J.Q., Farma J.M. (2017). Clinical Presentation and Staging of Melanoma. Cutaneous Melanoma: Etiology and Therapy.

[6] Perera E., Gnaneswaran N., Jennens R., Sinclair R. (2013). Malignant Melanoma. Healthcare.

[7] Shain A.H., Bastian B. (2016). From melanocytes to melanomas. Nat. Rev. Cancer.

[8] Soura E., Eliades P.J., Shannon K., Stratigos A.J., Tsao H. (2016). Hereditary melanoma: Update on syndromes and management: Genetics of familial atypical multiple mole melanoma syndrome. J. Am. Acad. Dermatol..

[9] Vaupel P., Kallinowski F., Okunieff P. (1989). Blood flow, oxygen and nutrient supply, and metabolic microenvironment of human tumors: A review. Cancer Res..

[10] Lartigau E., Randrianarivelo H., Avril M.F., Margulis A., Spatz A., Eschwège F., Guichard M. (1997). Intratumoral oxygen tension in metastatic melanoma. Melanoma Res..

[11] Evans S.M., Schrlau A.E., Chalian A.A., Zhang P., Koch C.J. (2006). Oxygen Levels in Normal and Previously Irradiated Human Skin as Assessed by EF5 Binding. J. Investig. Dermatol..

[12] Bedogni B., Powell M.B. (2009). Hypoxia, melanocytes and melanoma—Survival and tumor development in the permissive microenvironment of the skin. Pigment. Cell Melanoma Res..

[13] Carreau A., El Hafny-Rahbi B., Matejuk A., Grillon C., Kieda C. (2011). Why is the partial oxygen pressure of human tissues a crucial parameter? Small molecules and hypoxia. J. Cell. Mol. Med..

[14] Wang W., Winlove C.P., Michel C.C. (2003). Oxygen Partial Pressure in Outer Layers of Skin of Human Finger Nail Folds. J. Physiol..

[15] Trojan S., Piwowar M., Ostrowska B., Laidler P., Kocemba-Pilarczyk K.A. (2018). Analysis of Malignant Melanoma Cell Lines Exposed to Hypoxia Reveals the Importance of PFKFB4 Overexpression for Disease Progression. Anticancer Res..

[16] Minchenko O.H., Ogura T., Opentanova I.L., Minchenko D.O., Esumi H. (2005). Splice isoform of 6-phosphofructo-2-kinase/fructose-2,6-bisphosphatase-4: Expression and hypoxic regulation. Mol. Cell. Biochem..

[17] Potter C., Harris A.L. (2004). Hypoxia Inducible Carbonic Anhydrase IX, Marker of Tumour: Hypoxia, Survival Pathway and Therapy Target. Cell Cycle.

[18] Kaluz S., Kaluzová M., Liao S.-Y., Lerman M., Stanbridge E.J. (2009). Transcriptional control of the tumor- and hypoxia-marker carbonic anhydrase 9: A one transcription factor (HIF-1) show?. Biochim. Biophys. Acta BBA Bioenergy.

[19] Benej M., Pastorekova S., Pastorek J. (2014). Carbonic anhydrase IX: Regulation and role in cancer. Subcell. Biochem..

[20] Shin H.-J., Rho S.B., Jung D.C., Han I.-O., Oh E.-S., Kim J.-Y. (2011). Carbonic anhydrase IX (CA9) modulates tumor-associated cell migration and invasion. J. Cell Sci..

[21] Pastorekova S., Gillies R.J. (2019). The role of carbonic anhydrase IX in cancer development: Links to hypoxia, acidosis, and beyond. Cancer Metastasis Rev..

[22] Chafe S.C., McDonald P.C., Saberi S., Nemirovsky O., Venkateswaran G., Burugu S., Gao D., Delaidelli A., Kyle A.H., Baker J.H.E. (2019). Targeting Hypoxia-Induced Carbonic Anhydrase IX Enhances Immune-Checkpoint Blockade Locally and Systemically. Cancer Immunol. Res..

[23] Andreucci E., Peppicelli S., Carta F., Brisotto G., Biscontin E., Ruzzolini J., Bianchini F., Biagioni A., Supuran C.T., Calorini L. (2017). Carbonic anhydrase IX inhibition affects viability of cancer cells adapted to extracellular acidosis. J. Mol. Med..

[24] Federici C., Lugini L., Marino M.L., Carta F., Iessi E., Azzarito T., Supuran C.T., Fais S. (2016). Lansoprazole and carbonic anhydrase IX inhibitors sinergize against human melanoma cells. J. Enzym. Inhib. Med. Chem..

[25] Hsin M.-C., Hsieh Y.-H., Hsiao Y.-H., Chen P.-N., Wang P.-H., Yang S.-F. (2021). Carbonic Anhydrase IX Promotes Human Cervical Cancer Cell Motility by Regulating PFKFB4 Expression. Cancers.

[26] Manzano A., Pérez J., Nadal M., Estivill X., Lange A., Bartrons R. (1999). Cloning, expression and chromosomal localization of a human testis 6-phosphofructo-2-kinase/fructose-2,6-bisphosphatase gene. Gene.

[27] Chesney J., Clark J., Lanceta L., Trent J.O., Telang S. (2015). Targeting the sugar metabolism of tumors with a first-in-class 6-phosphofructo-2-kinase (PFKFB4) inhibitor. Oncotarget.

[28] Kotowski K., Rosik J., Machaj F., Supplitt S., Wiczew D., Jabłońska K., Wiechec E., Ghavami S., Dzięgiel P. (2021). Role of PFKFB3 and PFKFB4 in Cancer: Genetic Basis, Impact on Disease Development/Progression, and Potential as Therapeutic Targets. Cancers.

[29] Hasemann C.A., Istvan E.S., Uyeda K., Deisenhofer J. (1996). The crystal structure of the bifunctional enzyme 6-phosphofructo-2-kinase/fructose-2,6-bisphosphatase reveals distinct domain homologies. Structure.

[30] Bartrons R., Simon H., Rodríguez-García A., Castaño E., Navarro-Sabate A., Manzano A., Martinez-Outschoorn U. (2018). Fructose 2,6-Bisphosphate in Cancer Cell Metabolism. Front. Oncol..

[31] Chen L., Zhang Z., Hoshino A., Zheng H., Morley M., Arany Z., Rabinowitz J.D. (2019). NADPH production by the oxidative pentose-phosphate pathway supports folate metabolism. Nat. Metab..

[32] Shi L., Pan H., Liu Z., Xie J., Han W. (2017). Roles of PFKFB3 in cancer. Signal Transduct. Target. Ther..

[33] Ros S., Schulze A. (2013). Balancing glycolytic flux: The role of 6-phosphofructo-2-kinase/fructose 2,6-bisphosphatases in cancer metabolism. Cancer Metab..

[34] Chesney J., Clark J., Klarer A.C., Imbert-Fernandez Y., Lane A.N., Telang S. (2014). Fructose-2,6-Bisphosphate synthesis by 6-Phosphofructo-2-Kinase/Fructose-2,6-Bisphosphatase 4 (PFKFB4) is required for the glycolytic response to hypoxia and tumor growth. Oncotarget.

[35] Ros S., Santos C.R., Moco S., Baenke F., Kelly G., Howell M., Zamboni N., Schulze A. (2012). Functional Metabolic Screen Identifies 6-Phosphofructo-2-Kinase/Fructose-2,6-Biphosphatase 4 as an Important Regulator of Prostate Cancer Cell Survival. Cancer Discov..

[36] Yi M., Ban Y., Tan Y., Xiong W., Li G., Xiang B. (2019). 6-Phosphofructo-2-kinase/fructose-2,6-biphosphatase 3 and 4: A pair of valves for fine-tuning of glucose metabolism in human cancer. Mol. Metab..

[37] Telang S., Yalcin A., Clem A.L., Bucala R., Lane A.N., Eaton J.W., Chesney J. (2006). Ras transformation requires metabolic control by 6-phosphofructo-2-kinase. Oncogene.

[38] Kessler R., Fleischer M., Springsguth C., Bigl M., Warnke J.-P., Eschrich K. (2019). Prognostic Value of PFKFB3 to PFKFB4 mRNA Ratio in Patients With Primary Glioblastoma (IDH-Wildtype). J. Neuropathol. Exp. Neurol..

[39] Kumar P., Sharoyko V.V., Spegel P., Gullberg U., Mulder H., Olsson I., Ajore R. (2013). The transcriptional co-repressor myeloid translocation gene 16 inhibits glycolysis and stimulates mitochondrial respiration. PLoS ONE.

[40] Minchenko O., Opentanova I., Minchenko D., Ogura T., Esumi H. (2004). Hypoxia induces transcription of 6-phosphofructo-2-kinase/fructose-2,6-biphosphatase-4 gene via hypoxia-inducible factor-1α activation. FEBS Lett..

[41] Zhang H., Lu C., Fang M., Yan W., Chen M., Ji Y., He S., Liu T., Chen T., Xiao J. (2016). HIF-1α activates hypoxia-induced PFKFB4 expression in human bladder cancer cells. Biochem. Biophys. Res. Commun..

[42] Dasgupta S., Rajapakshe K., Zhu B., Nikolai B., Yi P., Putluri N., Choi J.M., Jung S.Y., Coarfa C., Westbrook T.F. (2018). Metabolic enzyme PFKFB4 activates transcriptional coactivator SRC-3 to drive breast cancer. Nature.

[43] Wang Q., Zeng F., Sun Y., Qiu Q., Zhang J., Huang W., Huang J., Huang X., Guo L. (2017). Etk Interaction with PFKFB4 Modulates Chemoresistance of Small-cell Lung Cancer by Regulating Autophagy. Clin. Cancer Res..

[44] Strohecker A.M., Joshi S.P., Possemato R., Abraham R.T., Sabatini D.M., White E. (2015). Identification of 6-phosphofructo-2-kinase/fructose-2,6-bisphosphatase as a novel autophagy regulator by high content shRNA screening. Oncogene.

[45] Sittewelle M., Kappès V., Lécuyer D., Monsoro-Burq A.H. (2021). The glycolysis regulator PFKFB4 interacts with ICMT and activates RAS/AKT signaling-dependent cell migration in melanoma. Preprint.

[46] Kocemba-Pilarczyk K.A., Ostrowska B., Trojan S., Aslan E., Kusior D., Lasota M., Lenouvel C., Dulińska-Litewka J. (2018). Targeting the hypoxia pathway in malignant plasma cells by using 17-allylamino-17-demethoxygeldanamycin. Acta Biochim. Pol..

[47] Ye J., Coulouris G., Zaretskaya I., Cutcutache I., Rozen S., Madden T.L. (2012). Primer-BLAST: A tool to design target-specific primers for polymerase chain reaction. BMC Bioinform..

[48] Kocemba-Pilarczyk K.A., Trojan S., Ostrowska B., Lasota M., Dudzik P., Kusior D., Kot M. (2020). Influence of metformin on HIF-1 pathway in multiple myeloma. Pharmacol. Rep..

[49] Humphrey W., Dalke A., Schulten K. (1996). VMD: Visual molecular dynamics. J. Mol. Graph..

